# MCPIP1 Elicits a Therapeutic Effect on Cervical Cancer by Facilitating XIAP mRNA Decay via Its Endoribonuclease Activity

**DOI:** 10.3390/ijms251910285

**Published:** 2024-09-24

**Authors:** Junyun Luo, Ling He, Yanxia Guo, Junzhi Wang, Hui Liu, Zhaoyong Li

**Affiliations:** 1 Hunan Provincial Key Laboratory of Medical Virology, Institute of Pathogen Biology and Immunology of College of Biology, Hunan University, Changsha 410082, China; 2 School of Biomedical Sciences, Hunan University, Changsha 410082, China; 3 State Key Laboratory of Chemical Oncogenomics, Tsinghua Shenzhen International Graduate School, Tsinghua University, Shenzhen 518055, China

**Keywords:** cervical cancer, MCPIP1, endonuclease, XIAP, cell apoptosis

## Abstract

Cervical cancer is the fourth most common malignancy in women globally. Chemotherapies, targeted therapies, and immunotherapies in the treatment of cervical cancer are usually accompanied by effective and adverse effects. Therefore, finding other efficient and accurate molecular targets remains essential to improve the treatment benefits of cervical cancer patients. MCPIP1 (monocyte chemoattractant protein-induced protein 1) is a kind of endonuclease with a CCCH zinc finger domain and a PilT-N-terminal (PIN) domain, and its function in cervical cancer is unknown. We found that MCPIP1 inhibits cell proliferation and promotes cell apoptosis of cervical cancer. Additionally, MCPIP1 suppresses mRNA and protein expression of the apoptotic inhibitor XIAP by decreasing its mRNA stability. Mechanically, MCPIP1 binds to the XIAP mRNA via its CCCH zinc finger domain and degrades the XIAP mRNA via the endonuclease activity coming from its PIN domain. Our study clarifies that MCPIP1 promotes cervical cancer cell apoptosis by suppressing the expression of XIAP, thereby impeding cervical cancer progression. Moreover, targeted delivery of MCPIP1 with engineered *Salmonella typhimurium* leads to tumor growth retardation in the HeLa xenograft tumor model in mice. Therefore, our study may provide a theoretical basis for formulating clinical treatment strategies for cervical cancer.

## 1. Introduction

Despite highly preventable vaccines and cancer screening programs, cervical cancer has become the fourth most common female cancer and cause of cancer death in women worldwide [1]. Although surgery, chemotherapy, and radiation therapy have been developed as the primary treatment methods for early cervical cancer, it remains a challenge to cure women with metastatic or recurrent disease [2]. Additionally, the adverse side effects of chemo- or radiotherapy on patients may lead to other disease risks. Consequently, biotherapy, such as targeted therapy or immunotherapy, has recently been focused on as an essential tool against cervical cancer [3,4]. Therefore, the investigation of the pathogenesis of cervical cancer and identification of critical molecular targets is of great significance for the effective treatment of cervical cancer.

MCPIP1, also known as regulatory RNase-1 (Regnase-1) [5], is the most well-studied and well-described member of MCPIP family proteins, which share a common feature: they all contain a PIN domain and a CCCH-type zinc finger domain. The PIN domain, which is a conserved domain located in the N-terminal part of MCPIP1 and confers it the endoribonuclease activity, contains a catalytic pocket composed of conversed acidic residues such as Asp141, Asp225, Asp226, and Asp244 [6]. Single-mutation analysis demonstrated that the transition of the aspartic acid into asparagine at 141 positions (D141N) of MCPIP1 led to a nearly complete loss of its RNase activity [7]. The CCCH zinc finger domain, which prefers to bind RNA by recognizing specific sequences or secondary structures [8], contributes to the recognition and direct binding of MCPIP1 to mRNAs [9]. MCPIP1 has a high affinity for single-stranded RNA (ssRNA), and it generally binds to mRNA in the 3′ untranslated region (UTR) and degrades the mRNA in the stem-loop structure [10,11].

MCPIP1 is initially described as one of the negative regulatory factors in the inflammatory response during the early phase of inflammation [7,12]. However, emerging studies have demonstrated that the function of MCPIP1 is closely related to tumor-related processes. MCPIP1 may function as a tumor suppressor in clear cell renal cell carcinoma (ccRCC) [13], leukemia [14], squamous cell carcinoma [15], melanoma [16], and triple-negative breast cancer [17], whereas it plays an oncogenic role in glioma [18]. For instance, MCPIP1 inhibits the viability and proliferation of ccRCC and tumor growth in vivo [13]. Moreover, it suppresses tumor vascularization in vivo through endonuclease activity. Mechanically, MCPIP1 degrades the mRNA of C/EBPβ, a transcription factor regulating the transcription of SDF-1, which cooperates with its receptor, CXCR4, to promote the expression of proangiogenic factors, such as VEGF. Nonetheless, it remains largely unknown about the function of MCPIP1 in cervical cancer. Here, we found that MCPIP1 inhibits cell proliferation and promotes cervical cancer cell apoptosis. Additionally, MCPIP1 suppresses mRNA and protein expression of the apoptotic inhibitor, XIAP, by decreasing its mRNA stability. Mechanically, MCPIP1 may bind to the XIAP mRNA via its CCCH zinc finger domain and degrade the XIAP mRNA via the endonuclease activity coming from its PIN domain. Our study would clarify that MCPIP1 promotes cervical cancer cell apoptosis by suppressing the expression of XIAP, thereby impeding cervical cancer progression.

Targeted delivery of exogenous MCPIP1 into tumor microenvironments (TMEs) could help us to elucidate its functional role in cervical cancer. Compared with conventional drug delivery systems, attenuated bacteria, including *Salmonella typhimurium*, *E. coli*, and *Bifidobacteria*, have been proven to be excellent delivery vectors due to their high tumor specificity, deep tissue penetration, and remote control capacity [19,20]. Engineered bacteria armed with oncolytic payloads have been reported to enhance cancer suppression in numerous cancer models; the overexpression of heterologous flagellin was proved to activate infiltrated immune cells in TMEs and promote the polarization of the M2 phenotype macrophage into an M1 phenotype, thus inhibiting cancer growth in colon cancer models [21]. Additionally, target delivery of cytolysin A (ClyA) into pancreatic cancer showed apparent destruction of cancer stromal cells and increased recruitment of immune cells in tumor tissues, leading to tumor shrinkage [22]. Moreover, engineered bacteria express imaging probes that enable visualized cancer theranostics [23]. In this research, we found that targeted delivery of MCPIP1 with engineered *S. Typhimurium* would suppress cervical cancer growth by inducing cancer cell apoptosis. Therefore, our study revealed the therapeutic potential of MCPIP1 against cervical cancer.

## 2. Results

### 2.1. MCPIP1 Promotes the Apoptosis of Cervical Cancer Cells

**Figure 1:**
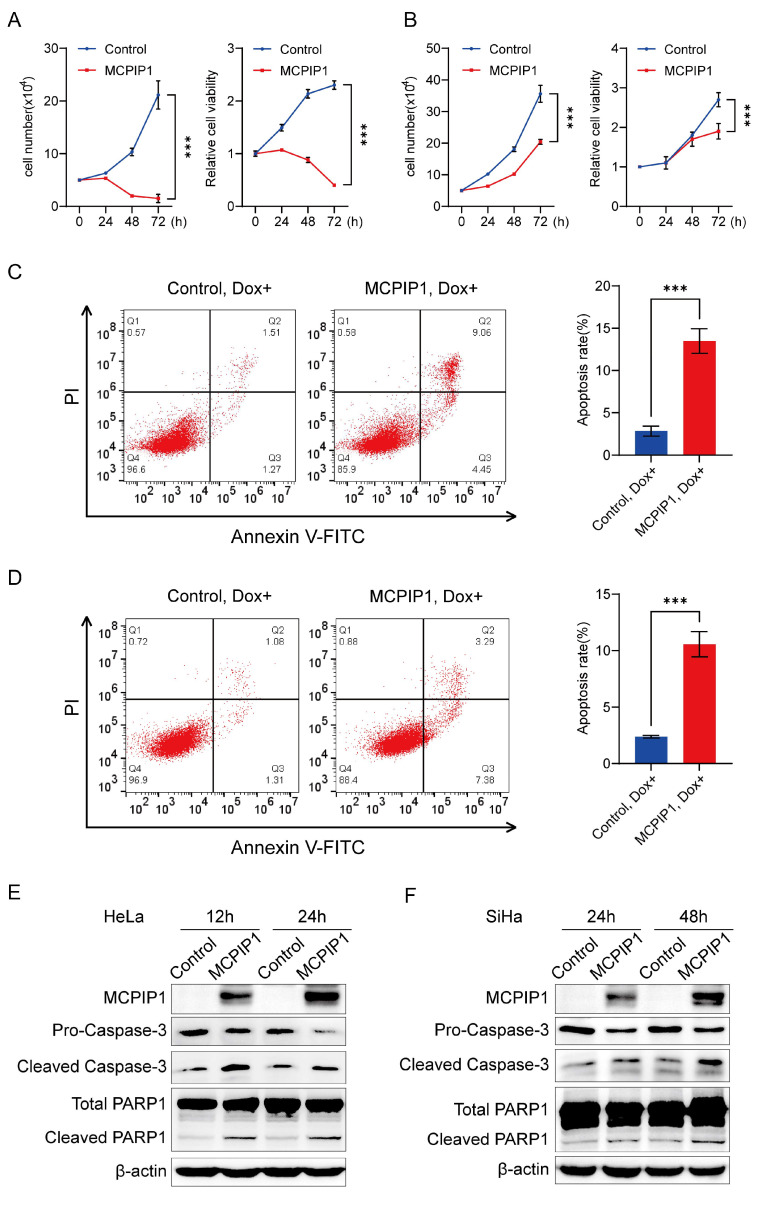
MCPIP1 promotes the apoptosis of cervical cancer cells. (**A**,**B**) The overexpression of MCPIP1 suppressed the proliferation of HeLa (**A**) and SiHa (**B**) cells, as assessed by the cell counting assay and CCK-8 assay. Data have been represented as mean ± SD. n = 3. (**C**,**D**) Flow cytometry assay for apoptosis detection of HeLa (**C**) and SiHa (**D**) cells infected with lentivirus of control or MCPIP1 and treated with 1 μg/mL doxycycline for 24 h and 48 h, respectively. Representative images were shown in the left panel. The apoptosis rates from three independent experiments were shown in the right panel. (**E**,**F**) Protein expression levels of MCPIP1, pro-caspase-3, cleaved caspase-3, total PARP1, and cleaved PARP1 were detected by Western blotting in HeLa cells (**E**) infected with lentivirus of control or MCPIP1 and treated with 1 μg/mL doxycycline for 12 and 24 h, respectively, or in SiHa cells (**F**) infected with lentivirus of control or MCPIP1 and treated with 1 μg/mL doxycycline for 24 and 48 h, respectively. β-actin was used as a loading control. *** *p* < 0.001.

MCPIP1 has been identified to function as a tumor suppressor or promoter in multiple cancers, but its function in cervical cancer remains unexplored. To investigate the impact of MCPIP1 on cervical cancer development, we first addressed whether MCPIP1’s overexpression affects cervical cancer cells’ growth. We infected the HeLa and SiHa cells with lentivirus of pLVX-TetOne-Puro (control) or pLVX-TetOne-MCPIP1 (MCPIP1) and induced the expression of MCPIP1 with 1 μg/mL doxycycline (Dox). Our cell counting assay and CCK-8 assay demonstrated that compared to the control group, MCPIP1 overexpression impeded the proliferation of both HeLa and SiHa cells (Figure 1A,B). Notably, compared to 0 and 24 h, the cell number of HeLa cells decreased at 48 and 72 h after Dox-induced expression of MCPIP1. Therefore, we speculate that MCPIP1 leads to HeLa cell apoptosis.

Apoptosis is a pivotal pathway in the pathogenesis of many diseases, including cervical cancer [24]. Pro-caspase in cells is cleaved into active forms of caspase during apoptosis, such as cleaved caspase-3, which functions as the executor of apoptosis. PARP1 is a substrate of activated caspases-3 in caspase-dependent apoptosis [25]. Cleavage of PARP1 protein by caspase enzymes leads to its inactivation, which prevents DNA damage repair and promotes caspase-mediated DNA fragmentation in apoptosis. To further ascertain our hypothesis that MCPIP1 regulates cervical cancer cell apoptosis, we induced the expression of MCPIP1 in HeLa and SiHa cells with doxycycline after lentivirus-mediated infection of the control or MCPIP1 and performed the flow cytometry assay. We found that compared to the control group, MCPIP1 overexpression results in a significantly higher apoptosis rate of cervical cells (Figure 1C,D). Furthermore, our Western blot analysis showed that the protein level of pro-caspase-3 decreased, but cleaved caspase-3 and cleaved PARP1 increased in MCPIP1-overexpressing HeLa and SiHa cells (Figure 1E,F), which further validated the pro-apoptotic role of MCPIP1 in cervical cancer cells.

### 2.2. The Apoptosis-Promoting Effect of MCPIP1 on Cervical Cancer Cells Depends on Its Endoribonuclease Activity

**Figure 2:**
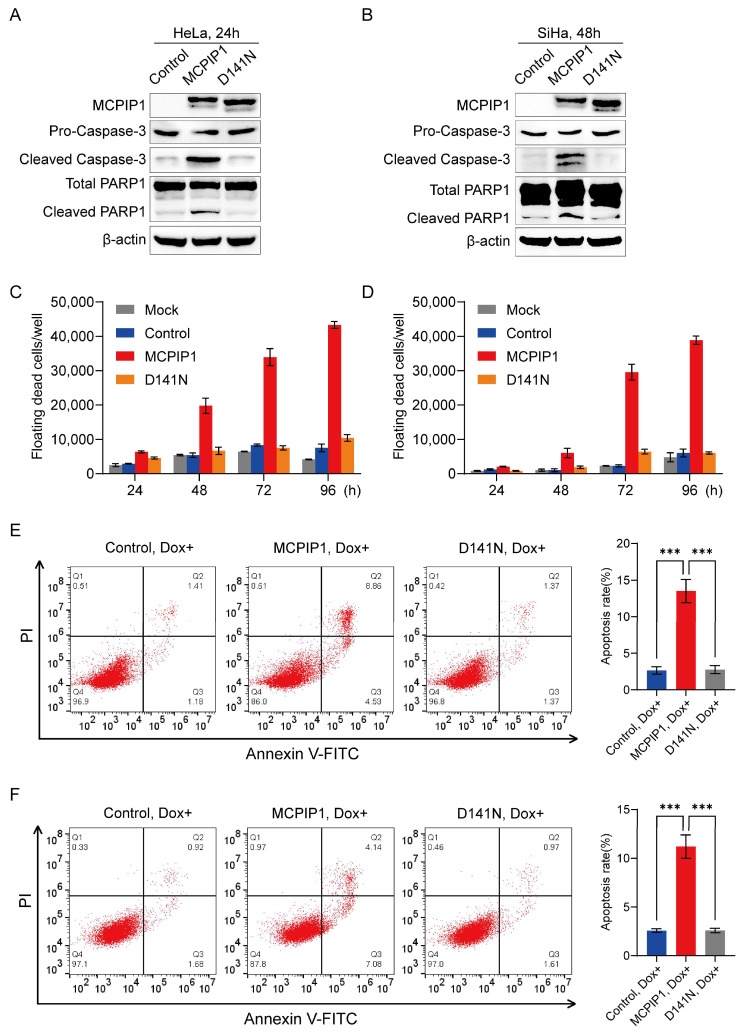
MCPIP1-mediated apoptosis in cervical cancer cells depends on its endonuclease activity. (**A**,**B**) Protein expression levels of MCPIP1, pro-caspase-3, cleaved caspase-3, total PARP1, and cleaved PARP1 were detected by Western blotting in HeLa (**A**) or SiHa (**B**) cells infected with lentivirus of control, MCPIP1, or D141N and treated with 1 μg/mL doxycycline for 24 and 48 h, respectively. β-actin was used as a loading control. (**C**,**D**) The number of floating cells (dead cells) were counted from the HeLa (**C**) and SiHa (**D**) cells after infection with control, MCPIP1, or D141N lentivirus for 24, 48, 72, and 96 h. (**E**,**F**) A flow cytometry assay was performed for apoptosis detection of HeLa (**E**) and SiHa (**F**) cells infected with lentivirus of control, MCPIP1, or D141N and treated with 1 μg/mL doxycycline for 24 and 48 h, respectively. Representative images and statistical analysis based on three independent experiments are shown. *** *p* < 0.001.

Utilizing its endoribonuclease activity, MCPIP1 plays multiple roles, such as the regulation of inflammation, apoptosis, adipogenesis, angiogenesis, and tumor progression [26,27]. To determine whether the endonuclease activity contributes to the apoptosis-promoting effect of MCPIP1 in cervical cancer cells, we mutated the pLVX-TetOne-MCPIP1 plasmid by changing the aspartic acid (D) at 141 of the PIN domain into asparagine (N), generating pLVX-TetOne-MCPIP1-D141N (D141N), which expresses mutant MCPIP1 that almost lost the endonuclease activity [7]. Our Western blotting results demonstrated that cleaved caspase-3 and cleaved PARP1 protein were enhanced in MCPIP1 but not D141N-overexpressing HeLa and SiHa cells (Figure 2A,B). Furthermore, MCPIP1 overexpression leads to more floating dead cells than the control and D141N overexpression in cervical cancer cells (Figure 2C,D). The flow cytometry assay also verified that MCPIP1 but not the control or D141N upregulated the percentages of apoptotic HeLa and SiHa cells (Figure 2E,F). Accordingly, the cell counting assay showed that Dox-induced MCPIP1 but not D141N expression suppressed the proliferation of HeLa and SiHa cells (Supplementary Figure S1A,B). In conclusion, our findings revealed that MCPIP1 facilitates cervical cancer cells’ apoptosis but restrains their growth, depending on its endoribonuclease activity.

### 2.3. MCPIP1 Promotes the Apoptosis of Cervical Cancer Cells via Suppressing XIAP Expression

**Figure 3:**
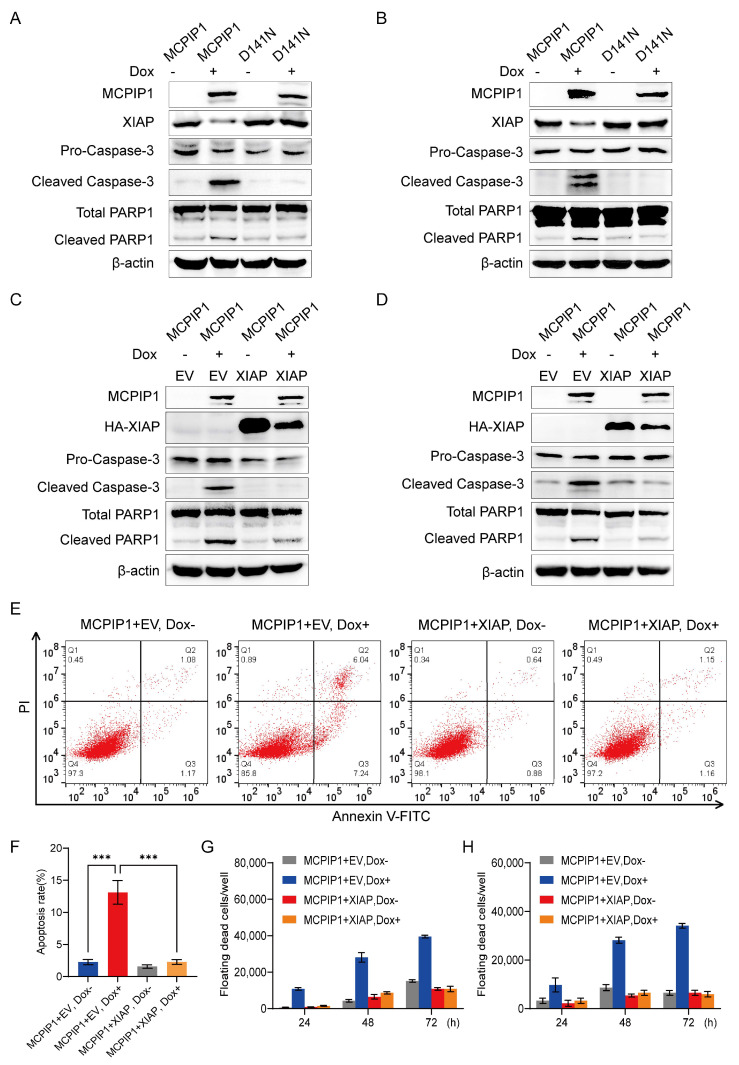
MCPIP1 facilitates cervical cancer cell apoptosis via XIAP. (**A**,**B**) Protein expression levels were detected by Western blotting in HeLa (**A**) or SiHa (**B**) cells, which were infected with a lentivirus of MCPIP1 or D141N and treated with or without 1 μg/mL doxycycline for 24 and 48 h, respectively. β-actin was used as a loading control. (**C**,**D**) HeLa (**C**) or SiHa (**D**) cells were co-infected with a lentivirus of MCPIP1 and pLVML-3×HA-MCS-IRES-Puro (EV) or pLVML-3xHA-XIAP (XIAP) and treated with or without 1 μg/mL doxycycline for 24 and 48 h, respectively. Then, Western blotting was performed with primary antibodies to MCPIP1, HA tag (HA-XIAP), pro-caspase-3, cleaved caspase-3, total PARP1, and cleaved PARP1. β-actin was used as a loading control. (**E**,**F**) A flow cytometry assay was utilized for the apoptosis assessment of HeLa cells co-infected with a lentivirus of MCPIP1 and XIAP and treated with or without 1 μg/mL doxycycline for 24 h. Representative images (**E**) and statistical analysis based on three independent experiments (**F**) were shown. (**G**,**H**) The floating dead cells were counted from the HeLa (**G**) and SiHa (**H**) cells after co-infection of the MCPIP1 and XIAP lentivirus and treated with or without 1 μg/mL doxycycline for 24, 48, and 72 h. *** *p* < 0.001.

Subsequently, we explore the underlying mechanism by which MCPIP1 regulates cervical cancer cell apoptosis. Inhibitors of apoptosis proteins (IAPs) are a family of proteins mainly known for their ability to bind and directly inhibit caspase activity [28]. As a result, IAPs have been identified to play extensive roles in cancer cell death, proliferation, invasion, and metastasis. Consequently, targeting IAP proteins has been considered an attractive strategy for cancer therapy. Among human IAP proteins, XIAP, IAP-1, and IAP-2 have well-characterized anti-apoptotic activity [29]. To examine whether MCPIP1 exerts its apoptosis-promoting effect by regulating the expression of IAP family members, we first induced the expression of MCPIP1 in HeLa and SiHa cells. Our RT-qPCR analysis showed that the overexpression of MCPIP1, but not D141N, leads to downregulating XIAP but not IAP-1 or IAP-2 mRNA levels, as well as decreased XIAP and increased cleaved PARP1 protein levels (Figure 3A,B and Figure S2A–D). Then, we addressed the function of XIAP in cervical cancer cell apoptosis.

As an IAP family member, XIAP is a highly conserved and widely expressed protein consisting of 497 amino acid residues and containing three BIR (baculovirus IAP repeat) domains, a UBA (ubiquitin-associated) domain, and a RING domain [30]. BIR2 and BIR3 domains of XIAP can directly inhibit the activation of caspase-3, -7, and -9, thus exerting its anti-apoptotic activity [29,30,31]. Our Western blotting assay demonstrated that XIAP knockdown in HeLa and SiHa cells led to increased expression of cleaved caspase-3 and cleaved PARP1 (Supplementary Figure S3A,B), verifying its apoptosis-resistant function in cervical cancer cells. However, if cervical cancer cells were co-infected with a lentivirus of MCPIP1 and XIAP, XIAP overexpression could attenuate the increase in cleaved caspase-3 and cleaved PARP1 protein activated by MCPIP1 overexpression (Figure 3C,D).

Additionally, our flow cytometry assay also demonstrated that the overexpression of MCPIP1 could enhance the apoptosis rate of HeLa and SiHa cells, but XIAP overexpression can impede its apoptosis-promoting function (Figure 3E,F and Figure S4A,B). Furthermore, forced expression of XIAP leads to fewer floating dead cells of HeLa and SiHa caused by MCPIP1 overexpression (Figure 3G,H). Moreover, XIAP overexpression also rescued the proliferation of HeLa and SiHa cells impeded by Dox-induced MCPIP1 overexpression (Supplementary Figure S5A,B). These findings suggested that XIAP can resist MCPIP1’s apoptosis-inducing effect in cervical cancer cells.

### 2.4. MCPIP1 Regulates the Expression of XIAP by Reducing Its mRNA Stability

**Figure 4:**
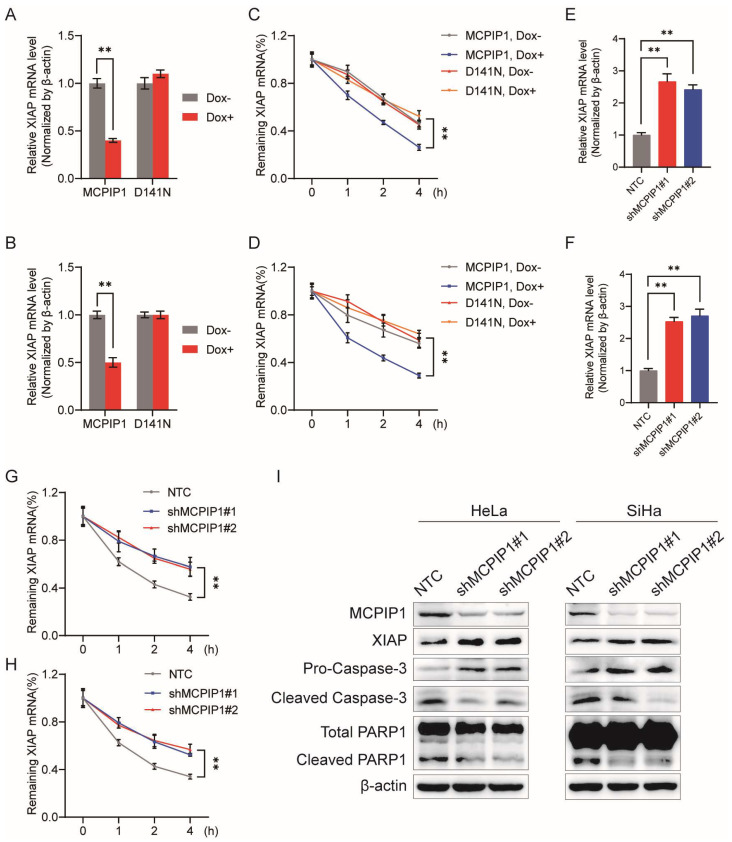
MCPIP1 attenuates XIAP mRNA’s stability, leading to its downregulated mRNA and protein expression. (**A**,**B**) HeLa (**A**) and SiHa (**B**) cells were infected with a lentivirus of MCPIP1 or D141N and treated with or without 1 μg/mL doxycycline for 24 and 48 h, respectively. Then, the XIAP mRNA levels were determined by RT-qPCR analysis. (**C**,**D**) HeLa (**C**) and SiHa (**D**) cells were infected with MCPIP1 or D141N lentivirus and treated with or without 1 μg/mL doxycycline for 24 h and 48 h, respectively. Then, cells were treated with 5 μg/mL actinomycin D for 0, 1, 2, and 4 h and collected to detect the remaining mRNAs of XIAP by RT-qPCR. (**E**,**F**) HeLa (**E**) and SiHa (**F**) cells were infected with the lentivirus of NTC or shRNAs to MCPIP1 (shMCPIP1#1 and shMCPIP1#2) for 48 h, and then the XIAP mRNA levels were determined by RT-qPCR analysis. (**G**,**H**) HeLa (**G**) and SiHa (**H**) cells were infected with the lentivirus of NTC, shMCPIP1#1, or shMCPIP1#2 for 48 h. Then, cells were treated with 5 μg/mL actinomycin D for 0, 1, 2, and 4 h and collected to determine the remaining mRNAs of XIAP by RT-qPCR. (**I**) Protein expression levels of MCPIP1, XIAP, pro-caspase-3, cleaved caspase-3, total PARP1, and cleaved PARP1 were detected by Western blotting in HeLa or SiHa cells, which were infected with the lentivirus of NTC, shMCPIP1#1, or shMCPIP1#2 for 48 h. β-actin was used as a loading control. ** *p* < 0.01.

Because MCPIP1 downregulates both mRNA and protein levels of XIAP, we further investigated the regulatory mechanism of MCPIP1 on XIAP expression. Considering the endonuclease function of MCPIP1, we speculated whether MCPIP1 affects XIAP mRNA degradation by its RNase activity. As expected, similar to IL-17RA mRNA that has been reported to be degraded by MCPIP1 via its endoribonuclease activity [12] (Supplementary Figure S2E,F), our RT-qPCR analysis showed that the Dox-induced expression of MCPIP1 but not D141N leads to downregulating XIAP mRNA levels in HeLa and SiHa cells (Figure 4A,B), which was in accordance with MCPIP1’s regulatory effect on XIAP’s protein (Figure 3A,B). Subsequently, we examined MCPIP1’s impact on XIAP’s mRNA stability. As shown in Figure 4C,D, a Dox-induced expression of MCPIP1 but not D141N leads to a shorter half-life of XIAP mRNA in HeLa and SiHa cells. We also infected the cervical cancer cells with a lentivirus of MCPIP1 shRNAs and determined the XIAP’s mRNA levels by RT-qPCR analysis. It was shown that MCPIP1 knockdown results in the upregulation of XIAP mRNA levels in HeLa and SiHa cells (Figure 4E,F). The RNA stability detection assay also demonstrated that compared to the NTC (pLKO.1) control, MCPIP1 knockdown leads to a longer half-life of XIAP mRNA (Figure 4G,H). These findings validate that MCPIP1 can inhibit the expression of XIAP by promoting its mRNA decay. As a result, increased protein expression levels of XIAP, as well as decreased protein levels of cleaved caspase-3 and cleaved PARP1, can be observed in HeLa or SiHa cells with MCPIP1 knockdown (Figure 4I). Therefore, MCPIP1 may decrease the mRNA and protein expression of XIAP by attenuating its mRNA stability, further leading to increased cell apoptosis.

### 2.5. The Regulation of XIAP by MCPIP1 Depends on Its ZF Domain

**Figure 5:**
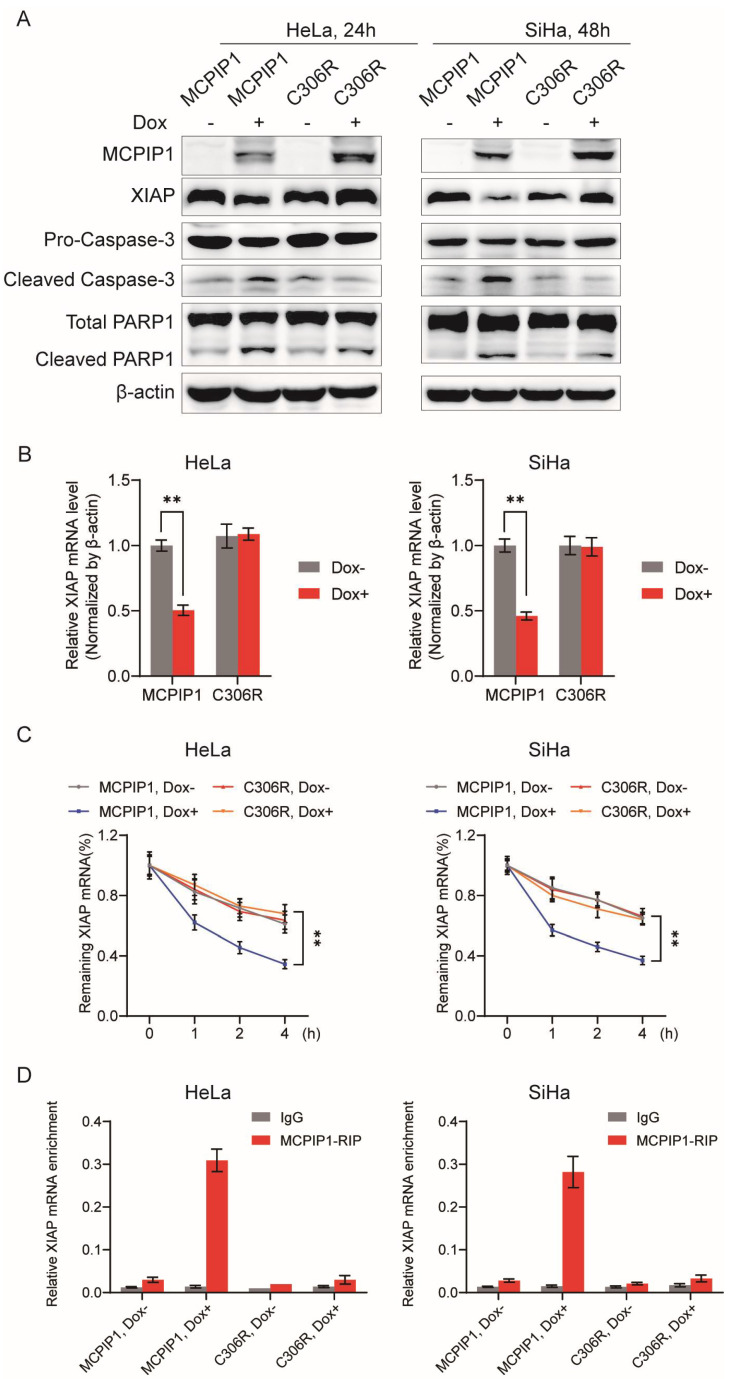
The ZF domain of MCPIP1 mediates its binding to XIAP mRNA and contributes to XIAP mRNA decay. HeLa and SiHa cells were infected with MCPIP1 or C306R lentivirus and treated with or without 1 μg/mL doxycycline for 24 and 48 h, respectively. (**A**) Protein expression levels of MCPIP1, XIAP, pro-caspase-3, cleaved caspase-3, total PARP1, and cleaved PARP1 were detected by Western blotting. β-actin was used as a loading control. (**B**) The XIAP mRNA expression was determined by RT-qPCR analysis. (**C**) HeLa or SiHa cells were further treated with 5 μg/mL actinomycin D for 0, 1, 2, and 4 h and collected to determine the remaining mRNAs of XIAP by RT-qPCR. (**D**) After an RIP assay with an antibody to MCPIP1 or IgG control, the enrichment of XIAP mRNA by MCPIP1 or C306R protein was examined by RT-qPCR. ** *p* < 0.01.

Due to the CCCH zinc finger domain of MCPIP1 being usually responsible for its binding to mRNAs [9], we then tested whether the regulatory function of MCPIP1 to XIAP’s mRNA stability relies on its zinc finger domain. We infected HeLa and SiHa cells with the lentivirus of wild-type MCPIP1 or its mutant C306R that lost the ability to bind the XIAP mRNA and then treated the cells with or without doxycycline. The Western blotting results showed that MCPIP1 but not C306R overexpression reduced the protein levels of XIAP but increased cleaved caspase-3 and cleaved PARP1 in cervical cancer cells (Figure 5A). Similarly, the XIAP mRNA levels decreased in HeLa and SiHa cells with the induction of MCPIP1 but not C306R overexpression (Figure 5B). Consequently, C306R does not affect the mRNA or the protein expression level of XIAP in cervical cancer cells, which differs from MCPIP1.

Furthermore, the RNA stability detection assay showed that overexpressed MCPIP1 but not C306R leads to lower mRNA stability of XIAP (Figure 5C). Our RIP assay demonstrated that the XIAP mRNA was enriched by MCPIP1 but not its ZF domain mutant C306R (Figure 5D and Figure S6A,B), suggesting that the ZF domain contributes to the physical association between MCPIP1 and XIAP mRNA. Additionally, the flow cytometry assay showed that MCPIP1 but not C306R overexpression increased the apoptosis rate of HeLa and SiHa cells (Supplementary Figure S7A,B). Collectively, our findings indicate that the CCCH ZF domain of MCPIP1 is indispensable for its negative regulatory role in XIAP’s mRNA stability and its anti-apoptotic function.

### 2.6. MCPIP1, but Not Its D141N or C306R Mutant, Inhibits Cervical Cancer Tumor Growth In Vivo

**Figure 6:**
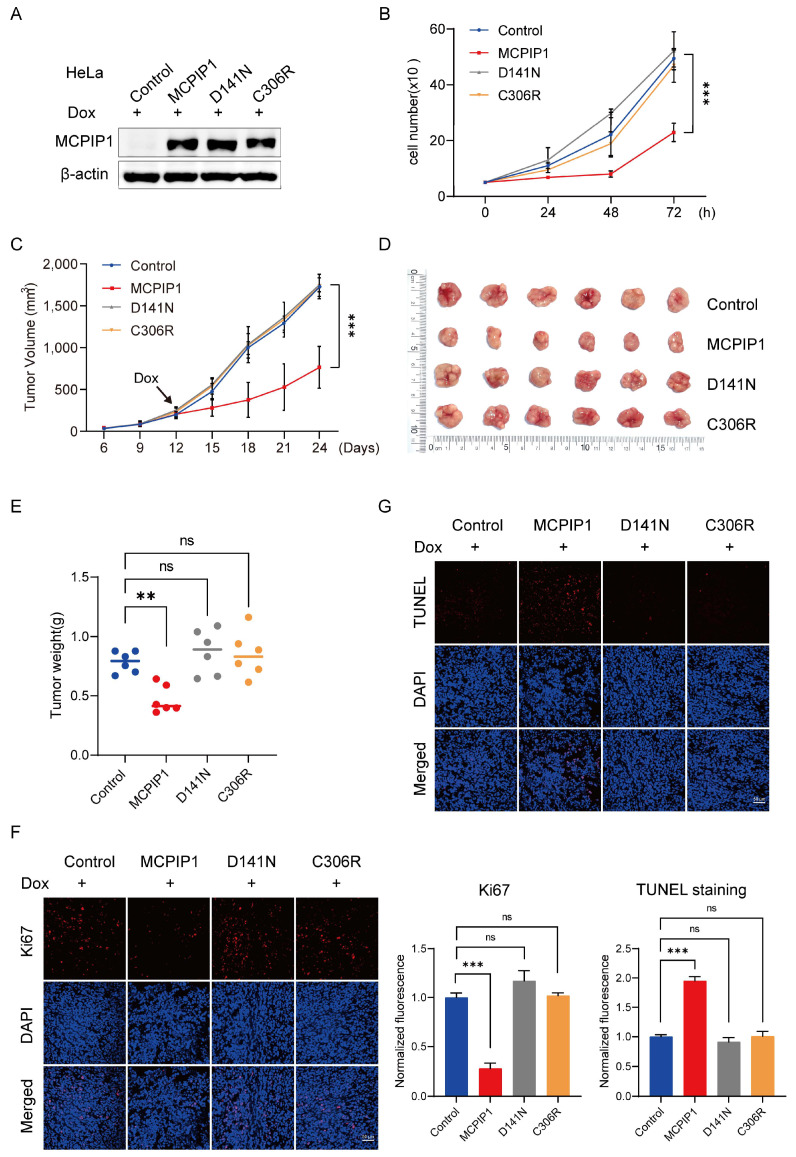
MCPIP1 promotes cell apoptosis but impedes tumor growth of cervical cancer in vivo. (**A**) A Western blotting assay showed MCPIP1 protein levels in HeLa cells stably transduced with control, MCPIP1, D141N, or C306R. β-actin was used as a loading control. (**B**) A cell counting assay was performed to examine the proliferation of HeLa cells stably expressing control, MCPIP1, D141N, or C306R. (**C**–**E**) HeLa cells were subcutaneously injected into the nude mice (n = 6 per group). Twelve days after subcutaneous injection, all xenograft tumor models were intraperitoneally injected with doxycycline daily at 5 mg/kg to induce the expression of control, MCPIP1, D141N, or C306R. (**C**) Growth curve of xenografts in vivo. (**D**) A picture of xenografts isolated from indicated groups on day 24 after subcutaneous injection. (**E**) Tumor weights were measured in the indicated groups. (**F**,**G**) Contiguous tumor sections were stained with antibodies against the proliferation biomarker Ki67 (**F**) and TUNEL kit for apoptosis detection (**G**), and the nucleus was stained with 4′,6-diamidino-2-phenylindole (DAPI, blue); scale bar = 50 μm. ns: no significance, ** *p* < 0.01, *** *p* < 0.001.

To validate the anticancer effect of MCPIP1 on cervical cancer in vivo, we first established HeLa cell lines stably expressing the control, MCPIP1, D141N, or C306R (Figure 6A). The cell proliferation test results showed that compared to the control group, Dox-induced expression of MCPIP1, but not its mutants D141N or C306R, significantly reduced the cell growth rate (Figure 6B). Then, we injected four groups of HeLa cells subcutaneously into BALB/c nude mice. We induced the expression of MCPIP1 or its mutants in xenograft tumors by intraperitoneal injection of Dox when the tumors reached an average size of approximately 200 mm^3^. The successful inducible expression of MCPIP1 and its mutants in HeLa tumors was verified by a Western blotting assay (Supplementary Figure S8). Consistent with the results of in vitro cell experiments, compared to the control group, the growth rate of Dox-induced MCPIP1-expressing xenograft tumors, but not that of the D141N or C306R group, was significantly slowed down (Figure 6C). Similarly, on average, the Dox-induced MCPIP1-expressing mice had smaller tumor volumes and weights than in the control, D141N, and C306R groups (Figure 6D,E). We further examined the proliferation and apoptosis in tumor slices. The expression of cell proliferation marker Ki67 was lower in the MCPIP1 group than in the control, D141N, and C306R groups (Figure 6F). However, the TUNEL staining assay showed that more apoptotic cells were observed in the MCPIP1 group than in the other three groups (Figure 6G). To sum up, our findings suggest that MCPIP1, but not its mutant D141N or C306R, inhibits tumor growth but promotes the apoptosis of cervical cancer in vivo.

### 2.7. Engineered MCPIP1-Expressing Bacteria Induce Robust Anticancer Activity in Cervical Cancer

**Figure 7:**
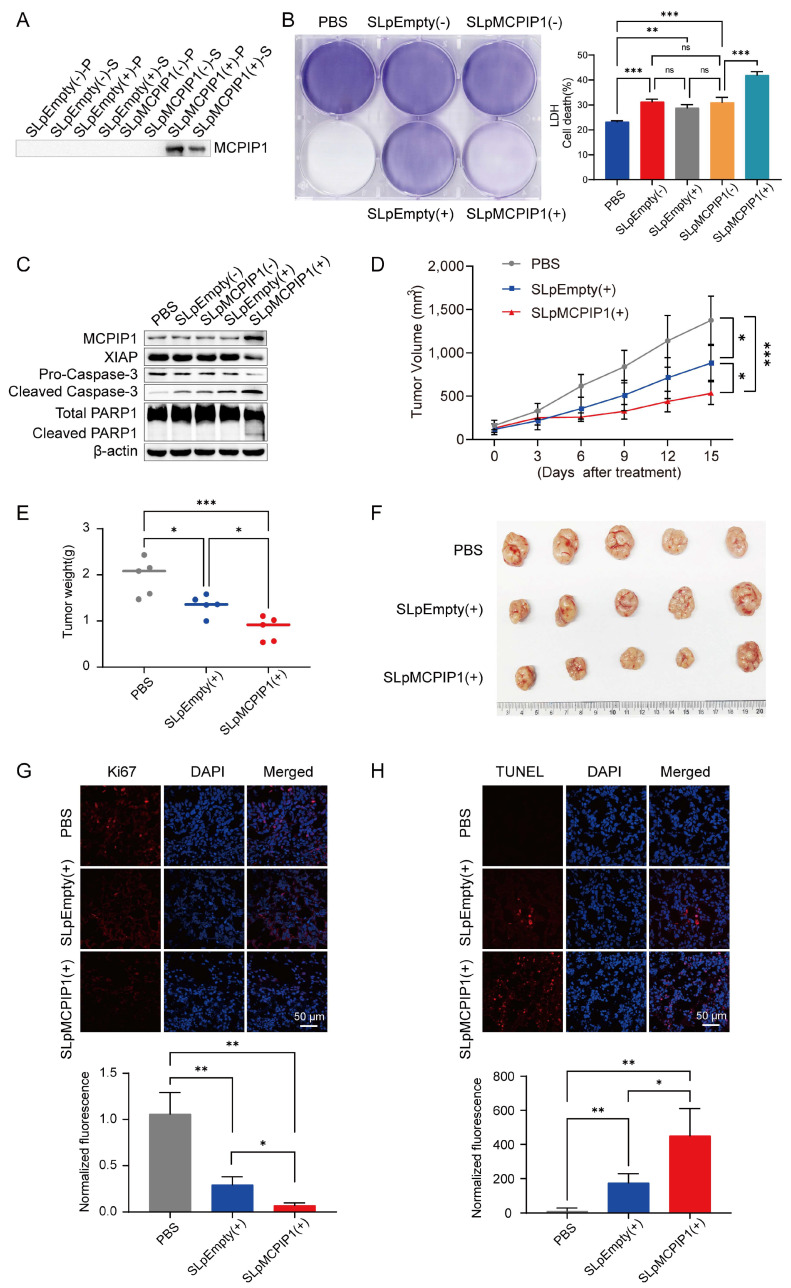
Anticancer efficiency of engineered MCPIP1-secreting *Salmonella typhimurium.* (**A**) Western blotting analysis to check the bacterial expression of MCPIP1 in vitro. Bacterial samples were prepared with (+) or without (−) 0.2% L-arabinose induction and separated into pellet (P) and supernatant (S) fractions. (**B**) Cell killing of HeLa by MCPIP1-expressing bacteria in vitro. Crystal violet staining was performed to check the viable cells, and the lactate dehydrogenase (LDH) released from HeLa cell co-culture with different treatments was determined with an LDH kit. The cell lysis of the positive control SLpMCPIP1 (+)-treated group was defined as 100%, n = 3. (**C**) HeLa cells were co-cultured with 1000 MOI SLpEmpty and SLpMCPIP1 with (+) or without (−) 24 h induction of 0.2% L-arabinose. All cell lysates were collected and detected by immunoblotting with the indicated antibodies. β-actin was used as an internal control. (**D**–**F**) Anticancer efficacy in the HeLa cancer model. BALB/c nude mice were implanted subcutaneously with HeLa cells. When the tumor reached 100–120 mm^3^, mice were treated with 1 × 10^7^ CFU-engineered bacteria (SLpEmpty or SLpMCPIP1) (n = 5 per group). (**D**) Average tumor growth in the HeLa cancer model. (**E**) Average tumor weight in the HeLa cancer model. (**F**) Image of the tumors from BALB/c nude mice at 15 dpi after different treatments. (**G**,**H**) Immunofluorescence staining for Ki67 and TUNEL. Contiguous HeLa tumor sections were stained with Ki67 (**G**) and TUNEL (**H**) antibodies, and nuclei were stained with DAPI (n = 3); scale bar = 50 μm. The average signal intensity was calculated and normalized to the PBS group. ns: no significance, * *p* < 0.05, ** *p* < 0.01, *** *p* < 0.001.

Attenuated *S. typhimurium* strains have been widely applied in various types of cancer (such as colon cancer, melanoma cancer, pancreatic cancer, etc.) [21,22,32,33,34,35,36]. Here, we used a targeted cancer therapy with attenuated *S. typhimurium* engineered to express MCPIP1 to treat HeLa cancer in a murine model. The MCPIP1 gene was cloned into the L-arabinose inducible pBAD expression system (termed pMCPIP1 or pEmpty as a control), as described previously [21,32]. Western blotting analysis revealed that the MCPIP1 protein was only detected in both the cell pellet and culture supernatant from pMCPIP1-carrying attenuated *S. typhimurium* (SLpMCPIP1) after L-arabinose induction (Figure 7A). To explore the cytotoxicity of SLpMCPIP1 in vitro, we performed crystal violet staining and lactate dehydrogenase release (LDH) assay experiments with the HeLa cell line. It was shown that SLpMCPIP1 (+) exhibited more effective cell killing than SLpEmpty (+), whereas considerable cell-killing effects were induced after SLpMCPIP1 (−), SLpEmpty (−), and SLpEmpty (+) treatment (Figure 7B). Furthermore, Western blot analysis showed that cleaved caspase-3 and cleaved PARP1 were increased after SLpMCPIP1 (+) treatment, whereas XIAP, which inhibits cell death, was decreased (Figure 7C).

To evaluate the antitumor effects of engineered *Salmonella typhimurium*, we intravenously injected the BALB/c nude mice implanted subcutaneously with HeLa tumors with PBS, SLpEmpty, or SLpMCPIP1 to examine the antitumor activity of engineered *S. typhimurium*. The volume and weight of tumors were decreased after SlpEmpty treatment, while they were greatly enhanced after SLpMCPIP1 (+) treatment (Figure 7D–F). Furthermore, Western blot analysis of excised HeLa tumor tissues demonstrated the overexpression of MCPIP1 in tumors treated with SLpMCPIP1 (+), and SLpMCPIP1 (+) further reduced XIAP but increased the expression of cleaved caspase-3 and cleaved PARP1 proteins compared with SLpEmpty (+) treatment (Supplementary Figure S9). Additionally, the cell proliferation marker Ki67 was more abundant in tumor tissues without bacterial colonization, and Ki67 was significantly decreased after SLpMCPIP1 (+) treatment compared with SLpEmpty (+) treatment (Figure 7G). Moreover, the cell death marker TUNEL was increased after bacterial treatment, and TUNEL signaling was stronger in the SLpMCPIP1 (+) group than in the SLpEmpty (+) group (Figure 7H). These results demonstrated that MCPIP1 could be used as a therapeutic target for cervical cancer, and attenuated *S. typhimurium* expressing MCPIP1 provides a new strategy for anticancer therapy.

## 3. Discussion

MCPIP1 is mainly located in the cytoplasm [37,38]. Using endonuclease activity in its N-terminal PIN domain, MCPIP1 can cause the degradation of some mRNAs, microRNAs, and viral RNAs to control their half-lives [26]. In addition to the protein-coding mRNAs that play essential functions in the inflammatory process, MCPIP1 also regulates some mRNAs related to the cancer process, thereby affecting cancer proliferation, angiogenesis, metastasis, and apoptosis [27,39]. Here, we found that MCPIP1 inhibits cell proliferation and promotes cell apoptosis, which was verified by enhanced apoptotic cells in flow cytometry assay and upregulated protein expression of cleaved caspase-3 and PARP1 in a Western blotting assay after MCPIP1 overexpression in cervical cancer cells (Figure 1). Furthermore, we confirmed that the regulatory effect of MCPIP1 on proliferation and apoptosis depends on its endoribonuclease activity because D141N, the MCPIP1 mutant whose endonuclease activity was almost destroyed, failed to increase the apoptotic and floating dead cells after its overexpression (Figure 2).

XIAP, an apoptosis inhibitor directly restraining the caspase activity, exhibits an apoptosis inhibitory function in cervical cancer cells because its deficiency leads to upregulated cleaved caspase-3 and PARP1 protein levels (Supplementary Figure S3). Our study identified XIAP as a target of MCPIP1 in exerting its apoptosis-promoting role in cervical cancer cells, which was supported by the findings that the forced expression of XIAP could reverse the pro-apoptotic effect of MCPIP1 on cervical cancer cells (Figure 3). Moreover, it was further revealed that MCPIP1 negatively regulates XIAP mRNA and protein expression by utilizing its endoribonuclease activity. Therefore, our research findings propelled us to conclude that XIAP mediates the apoptosis regulatory role of MCPIP1 in cervical cancer cells.

**Figure 8:**
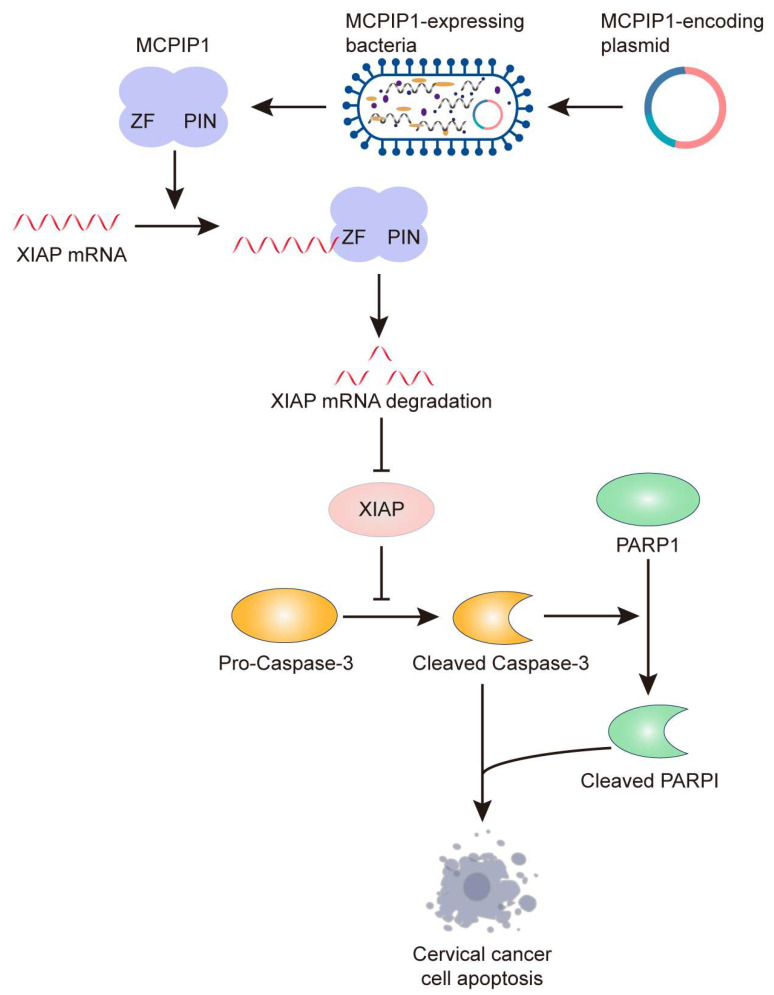
Schematic illustration of the potential mechanism by which MCPIP1 promotes cervical cancer apoptosis. MCPIP1 binds to XIAP mRNA by its ZF domain and degrades it by its endonuclease activity in the PIN domain. As a result, XIAP protein expression was restrained, leading to enhanced expression of cleaved caspase-3 and PARP1 that contributes to cervical cancer cell apoptosis. Through the XIAP/caspase/PARP1 axis, targeted delivery of MCPIP1 with engineered *Salmonella typhimurium* leads to increased cell apoptosis and inhibited tumor growth in the HeLa xenograft model in mice.

Homeostasis of mRNA levels is determined by the regulation of transcription or mRNA stability. Our study discovered that MCPIP1 weakens the mRNA stability of XIAP, depending on its endonuclease activity (Figure 4) because the D141N mutant of MCPIP1, whose RNase activity was nearly abolished, lost the regulatory effect on XIAP expression. Furthermore, wild-type MCPIP1 but not its C306R mutant could bind with XIAP mRNA and reduce XIAP’s mRNA level and stability, indicating that the normal function of the CCCH zinc finger domain of MCPIP1, which mediates the binding of it to XIAP mRNA, is essential for XIAP mRNA decay. Additionally, our in vivo experiments showed that MCPIP1, but not the D141N or C306R mutants, promotes tumor growth in mouse xenograft models, as well as the decreased staining of Ki67 and increased staining of TUNEL in tumor tissues. Collectively, we proposed a working model to show that MCPIP1 binds to XIAP mRNA via its ZF domain and facilitates its degradation by utilizing the endonuclease activity of the PIN domain (Figure 8). As a result, XIAP’s protein expression is suppressed in cervical cancer cells, leading to increased cell apoptosis and impediments to cervical cancer development.

Cervical cancer is one of the malignant tumors that seriously threaten the health of women worldwide [40]. The treatment for this cancer remains a challenge, with poor clinical prognosis and adverse effects of chemo- and radiotherapy in patients with advanced or recurrent disease [41]. Therefore, exploring new strategies with practical, accurate, and low side effects for cervical cancer treatment is still of great significance. In addition to the finding that MCPIP1 promotes apoptosis and inhibits cervical cancer progression in vivo and in vitro, our study found that the targeted delivery of exogenous MCPIP1 with engineered *S. typhimurium* showed significant inhibition of HeLa tumor growth. Histological analysis also revealed decreased Ki67 and increased TUNEL in tumor tissues in the SLpMCPIP1 (+) group compared with the SLpEmpty (+) group, which suggests that in situ expression of MCPIP1 could inhibit cell proliferation and promote cell apoptosis of cervical cancer. Therefore, our research uncovered the biomedical importance of MCPIP1 in the treatment of cervical cancer and provided a potential future therapeutic target for cervical cancer patients.

## 4. Materials and Methods

### 4.1. Cell Lines and Culture

Human cervical cancer cells, HeLa and SiHa, and human embryonic kidney (HEK293T) cells (CL-0005) were obtained from Procell (Wuhan, China). All cells were maintained in Dulbecco’s Modified Eagle’s Medium supplemented with 10% fetal bovine serum (FBS) and 1 % penicillin-streptomycin. Cells were maintained in a humidified atmosphere of 5% CO_2_ at 37 °C.

### 4.2. Plasmids Construction

Human MCPIP1 was amplified by PCR and cloned into the vector pLVX-TetOne-Puro via EcoRI/BamHI, generating MCPIP1 overexpression plasmids pLVX-TetOne-MCPIP1. To construct the XIAP overexpressing plasmid, the vector pLVML-3 × HA-MCS-IRES-Puro was used as the backbone for inserting a human XIAP coding sequence via XhoI/SpeI to generate pLVML-3xHA-XIAP. The vector pLKO.1-puro (Sigma, St. Louis, MO, USA) was utilized to create lentiviral shRNAs targeting human MCPIP1 and XIAP. The primers used to construct plasmids are listed in Supplementary Table S1.

To mutate the MCPIP1 coding sequence by changing the D (141) into N, the pLVX-TetOne-MCPIP1 vector DNA was used as a template for PCR amplification with forwarding (5′-GGTCATCAATGGGAGCAACGTGGCCATGAGCC-3′) and reverse (5′-TGCTCCCATTGATGACCACTGGTCTCAGGTCG-3′) primers. To mutate the MCPIP1 coding sequence by changing the C (306) into R, the pLVX-TetOne-MCPIP1 vector DNA was used as a template for PCR amplification with forwarding (5′-AAGCAGCcgcGTCCCTATGGAAGGAAATGCAC-3′) and reverse (5′-TAGGGACgcgGCTGCTTCCTGTGCTCCAAAGT-3′) primers. The PCR products were purified by a gel extraction kit, digested by Dpn I restriction enzymes, and transformed into the *Stbl3* to generate the pLVX-TetOne-MCPIP1-D141N (D141N) and pLVX-TetOne-MCPIP1-C306R (C306R) clones, respectively.

### 4.3. Lentivirus Package and Infection

The overexpressing or shRNA plasmids, packaging plasmid psPAX2, and envelope plasmid pMD2.G were co-transfected into HEK293T cells. The lentivirus was collected after 24 h and 48 h post-transfection. Then, lentiviruses expressing MCPIP1, its mutant plasmids, D141N and C306R, and its shRNA plasmids were used to infect HeLa or SiHa cells in the presence of polybrene.

### 4.4. Cell Proliferation Assay

The cell proliferation ability was examined by a cell counting assay. After overexpression or knockdown using lentivirus infection of genes for 24 h, HeLa or SiHa cells were seeded in flat-bottom 12-well plates at a density of 5 × 10^4^ cells per well and cultured for a total of 72 h. For every 24 h, the dead cells were stained with a tenth volume of 0.4% trypan blue, and the live cells were counted with a hemocytometer.

Cell viability was also determined using cell counting kit-8 (CCK-8 assay) (C0005, TragetMol, Shanghai, China). After overexpressing or knockdown lentivirus infection of genes for 24 h, HeLa or SiHa cells were inoculated onto 96-well plates at 5 × 10^3^ cells per well and cultured for another 12 h, allowing attaching the vessel. After 0, 24, 48, and 72 h, the culture medium was replaced with a 110 μL reaction buffer containing 10 μL CCK-8 reagents. Then, the cells were cultured for another 2 h, and the absorbance was measured at 450 nm with a Multiskan FC microplate reader (Thermo Scientific, Waltham, MA, USA). The relative cell viability was calculated as follows: relative cell viability = (OD450 value of MCPIP1 or control group − OD450 value of blank group) at a specified time point/(OD450 value of MCPIP1 or control group − OD450 value of blank group) at 0 h.

### 4.5. Flow Cytometry Assay

The flow cytometry assay was performed according to the manufacturer’s instructions of the Annexin V-FITC/PI Apoptosis Detection Kit (A211, Vazyme Biotech, Nanjing, China). HeLa or SiHa cells were plated in flat-bottom 6-well plates, infected with an overexpressed lentivirus and induced by Doxycycline. A total of 1 × 10^5^–5 × 10^5^ cells were collected, washed with cold PBS twice, and suspended in 100 μL of a binding buffer, followed by staining with 5 μL Annexin V-FITC and 5 μL PI for 10 min in the dark at room temperature. Then, 400 μL of a binding buffer was added into the incubation system, and the stained cell samples were immediately analyzed with a flow cytometer (CytoFLEX, Beckman, Brea, CA, USA). Untreated cells were used as negative controls, and the cells stained only with Annexin V-FITC or PI solution were applied for compensation for each trial. The results were analyzed using FlowJo software (version 10).

### 4.6. Western Blotting

Whole-cell lysates were prepared with a RIPA lysis buffer (P0013C, Beyotime, Shanghai, China) with protease inhibitors, and the protein concentration was quantified by a Modified Bradford Protein Assay Kit (C503041, Sangon Biotech, Shanghai, China). A total of 20 μg total protein of each cell lysate sample was separated on SDS-PAGE gels and then transferred to PVDF membranes (Millipore, Bedford, MA, USA). Membranes were processed using the ECL Western blotting protocol (P0018FM, Byeotime, Shanghai, China). The primary antibodies and concentrations used for Western blotting were MCPIP1 (1:1000, ab97910, Abcam, Cambridge, United Kingdom), XIAP (1:1000, 66800-1-Ig, Proteintech, Wuhan, China), full caspase-3 (1:1000, 19677-1-AP, Proteintech), cleaved caspase-3 (1:1000, 9661, cell signaling technology), PARP1 (1:1000, 13371-1-AP, Proteintech, Wuhan, China), HA tag (1:1000, 51064-2-AP, Proteintech, Wuhan, China), and β-actin (1:5000, 66009-1-Ig, Proteintech, Wuhan, China). Densitometry analysis was performed with ImageJ software (version 1.52a).

### 4.7. RT and Quantitative Real-Time PCR (RT-qPCR)

Total RNA from cell lines was extracted using TRIzol^TM^ reagent (ThermoFisher Scientific, Waltham, MA, USA) as per the manufacturer’s instructions. A total of 500 ng RNA was reverse transcribed into cDNA according to HiScript II Q RT SuperMix for qPCR (+gDNA wiper) instructions (R223, Vazyme, Nanjing, China). The quantitative real-time polymerase chain reaction (qPCR) was performed using the Bio-rad system (Hercules, CA, USA) with DNA-specific fluorescent dye SYBR Green I (Q711, Vazyme, Nanjing, China). The primers used for qPCR are listed in Supplementary Table S2. Expression levels of mRNA were normalized by that of the housekeeping gene β-actin. The relative expression levels were calculated using the method of 2^−ΔΔCT^.

### 4.8. RNA Immunoprecipitation Assay

Cells were fixed with 1% formaldehyde in a PBS buffer for 10 min at room temperature, and cross-linking was quenched with 0.125 M glycine for 5 min. Then, cell pellets were resuspended in the lysis buffer (50 mM Tris, 100 mM NaCl, 0.5% NP40, pH 8.0) containing a complete protease inhibitor cocktail (B14001, Bimake, Houston, TX, USA) and RNase inhibitor (R0102, Beyotime, Shanghai, China). Cell extracts were sonicated for 5 min with a sonicator (JY92-IIN, SCIENTZ, Ningbo, China), and a 100 μL sample of the supernatant was saved as the input. The remaining cell lysates were incubated with protein A/G magnetic beads (B23202, Bimake) conjugated with an antibody against normal rabbit IgG or MCPIP1 (ab97910, Abcam, Cambridge, UK) at 4 °C overnight. After washing at least three times with a lysis buffer, the immune complexes were mixed with TRIzol^TM^ reagent (ThermoFisher Scientific, Waltham, MA, USA) for RNA extraction according to the manufacturer’s instructions. RT-qPCR was applied to measure the RNAs enriched by protein.

### 4.9. Engineered Bacterial Strains

The human MCPIP1 gene (1866 base pairs) was amplified from the pUC57-MCPIP1 plasmid (Tsingke Biotechnology Co., Ltd., Beijing, China) using the following primers to engineer HA-MCPIP1-expressing bacteria: forward, CATGCCATGGCCTATCCGTACGATGTGCCGGATTATGCGATGAGCGGTCCGTGTGGTGAAAA; reverse, AGCTTTGTTTAAACTTATTCGCTCGGATGCTGGCTTTTATAGCTC. Amplified DNA was cut with *Nco*I and *Pme*I and used to replace Rluc8 directly at the same site in pBAD-pelB-Rluc8. The resulting plasmid was named pMCPIP1. The empty vector (control) was named pEmpty. Plasmids pEmpty and pMCPIP1 (both harboring an ampicillin resistance gene) were transferred into ΔppGpp *Salmonella typhimurium* by heat-shock transformation. The new strains (named SlpEmpty or SLpMCPIP1) were maintained in an ampicillin-containing medium and kept in a deep freezer at −80 °C as 25% glycerol stocks. The OD values of the cultured bacterial cells were measured at the absorbance of 600 nm (OD_600_). The number of SlpEmpty or SLpMCPIP1 was calculated by assuming that 1 OD_600_ represents 8 × 10^8^ colony-forming units (CFUs)/mL.

### 4.10. Crystalline Violet Staining

HeLa cells were seeded at a density of approximately 1 × 10^5^ cells per well in 6-well plates and incubated overnight. On the next day, the culture medium was removed and the cells were washed twice with DPBS and added to DMEM containing 10% FBS. A total of 0.2% L-arabinose-induced (+) or -uninduced (−) SlpEmpty and SLpMCPIP1 were used to infect the cells at 100 MOI (multiplicity of infection, bacteria: cell = 100:1). The cell supernatant was replaced with fresh medium every 24 h until 72 h of culture. Then, the cells were washed twice with DPBS, and 4% PFA was added to fix the cells at room temperature for 10 min. Then, the cells were washed twice with DPBS, and 1 mL of 0.1% crystal violet dye was added and incubated at room temperature for 30 min. The crystal violet solution was recovered and washed twice with DPBS, and photographs were obtained and analyzed.

### 4.11. Lactate Dehydrogenase Assay

HeLa cells pre-seeded into a 6-well plate (5 × 10^5^ cells per well) were co-incubated with 0.2% L-arabinose-induced (+) or -uninduced (−) SLpEmpty and SLpMCPIP1 (1000 MOI) for 24 h in DMEM with 3% FBS. The cell culture supernatants were analyzed using a CytoTox 96^®^ Non-Radioactive Cytotoxicity Assay kit (G1782, Promega Corporation, Madison, WI, USA) in accordance with the manufacturer’s instructions. The absorbance of the sample at 490 nm was detected using a microplate reader (SpectraMax M2/M2e, Molecular Devices, Silicon Valley, CA, USA). The cells treated with SLpMCPIP1 (+) were lysed to obtain the supernatant as the positive control and applied as the value of 100% in the calculation of cell killing.

### 4.12. Animal Xenograft Models

BALB/c nude mice (6–8 weeks of age, female) were purchased from Charles River Laboratories and kept under specific pathogen-free conditions in the animal care facility of Hunan University. All animal experiments were conducted in accordance with the guidelines of the Laboratory Animal Management Regulation with the approval of Hunan University. HeLa cells were infected with a lentivirus of EV, MCPIP1, D141N, or C306R. The stably transduced cells were generated by puromycin selection. Cells were trypsinized and washed with PBS. Live cells were counted with trypan blue exclusion, and 5 × 10^6^ cells were injected subcutaneously into the flanks of mice (n = 6). Tumor volume was measured once every three days after six days of inoculation. Doxycycline hyclate (Dox) was dissolved in water and utilized with a dose of 5 mg/kg of body weight. When the tumors reached an average size of approximately 200 mm^3^, Dox was administered by intraperitoneal injection once daily to induce the expression of MCPIP1 or its mutant in xenograft mice. Mice were euthanized before tumors exceeded 2000 mm^3^, and the tumor was dissected, photographed, and weighed.

For in vivo evaluation of anticancer activity with engineered MCPIP1-expressing *S. typhimurium*, subcutaneous HeLa tumor models were established via the subcutaneous injection of HeLa cells (5 × 10^6^) suspended in 100 μL of DPBS into the right flanks of BALB/c nude mice. When the tumor volume reached approximately 100–120 mm^3^, the mice were grouped randomly, treatments were applied, and the day was defined as 0 days post-implantation (dpi). The mice were intravenously injected with 100 μL of PBS containing 1 × 10^7^ CFU SlpEmpty or SLpMCPIP1. From 3 dpi onward, the mice were intraperitoneally injected with 200 μL of 40% L-arabinose to trigger MCPIP1 protein expression in situ. The tumor sizes were measured with a caliper every 3 days. The tumor volume (in cubic millimeters) was calculated using the following formula: (L × W × H)/2, where L is the length, W is the width, and H is the height of the tumor in millimeters. Mice were euthanized at 15 dpi; the weight and size of the tumors were recorded for further analysis.

### 4.13. Immunofluorescence and TUNEL Staining for Tumor Tissues

For immunofluorescence staining, tumors isolated from mice were immersed in 4% PFA at 4 °C for 2 h and then transferred into a 30% sucrose solution at 4 °C overnight, embedded in tissue fixation solution (OCT), and rapidly frozen at −80 °C. The OCT-embedded tumor sections (6 μm) were cut using a freezing microtome (LEICA CM1950, Weztlar, Germany) and mounted onto slides. The contiguous sections were stained with TUNNEL in accordance with the manufacturer’s instructions (C1089, Beyotime, Shanghai, China). A primary anti-Ki67 (27309-1-AP, Proteintech, Wuhan, China) antibody was incubated overnight at 4 °C and then washed and stained with a secondary antibody, donkey anti-rabbit 555 (A-31572, Invitrogen, Waltham, MA, USA). The slices were photographed with a two-photon laser scanning microscope (TI-E + A1RMP + N-STORM, Nikon, Tokyo, Japan).

### 4.14. Statistical Analysis

Statistical analysis was performed using Excel 2013 or GraphPad Prism 9.0 software. All results were shown as mean ± standard deviation from at least three independent experiments. A two-tailed Student’s *t*-test was performed for two groups, and a one-way ANOVA was used for multi-group comparison. For all analyses, *p* < 0.05 was considered to be statistically significant (ns: no significance, * *p* < 0.05, ** *p* < 0.01, *** *p* < 0.001).

## Data Availability

All data needed to evaluate the conclusions in this paper are present in this paper and/or the Supplementary Materials.

## Supplementary Materials

The following supporting information can be downloaded at https://www.mdpi.com/article/10.3390/ijms251910285/s1.
