# Supplementary material for: MCPIP1 Elicits a Therapeutic Effect on Cervical Cancer by Facilitating XIAP mRNA Decay via Its Endoribonuclease Activity
Source: Int J Mol Sci. 2024 Sep 24;25(19):10285. doi: 10.3390/ijms251910285 (PMC11477132; doi:10.3390/ijms251910285)
Supplement: Supplementary file 1 [file ijms-25-10285-s001.zip › ijms-3198279-supplementary.pdf]

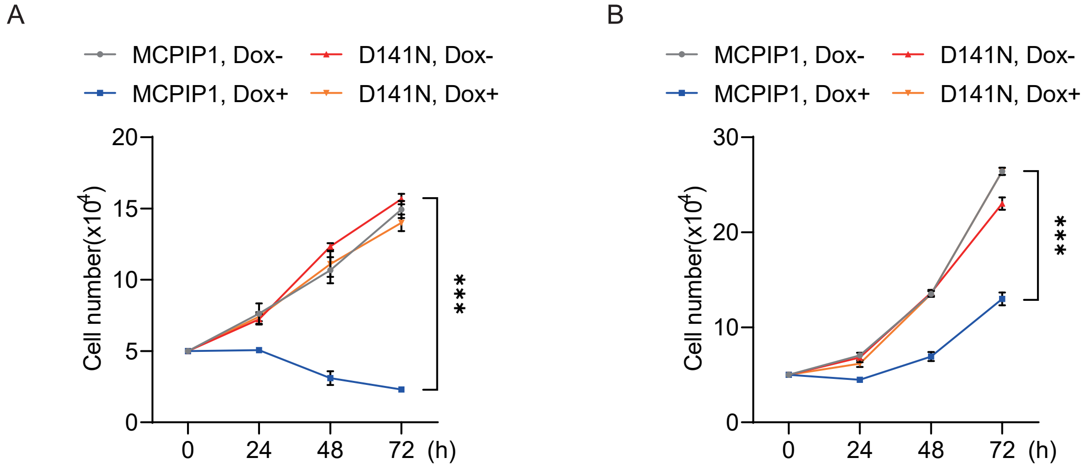

Supplementary Figure 1. MCPIP1, but not D141N, promotes cervical cancer cell proliferation. (**A**) HeLa cells were infected with lentivirus of MCPIP1 or D141N and treated with or without 1μg/ml doxycycline for 24 hrs. Then, the number of living cells was counted at 0, 24, 48, and 72 hrs. (**B**) SiHa cells were infected with lentivirus of MCPIP1 or D141N and treated with or without 1μg/ml doxycycline for 48h. Then, the number of living cells was counted at 0, 24, 48, and 72 hrs.

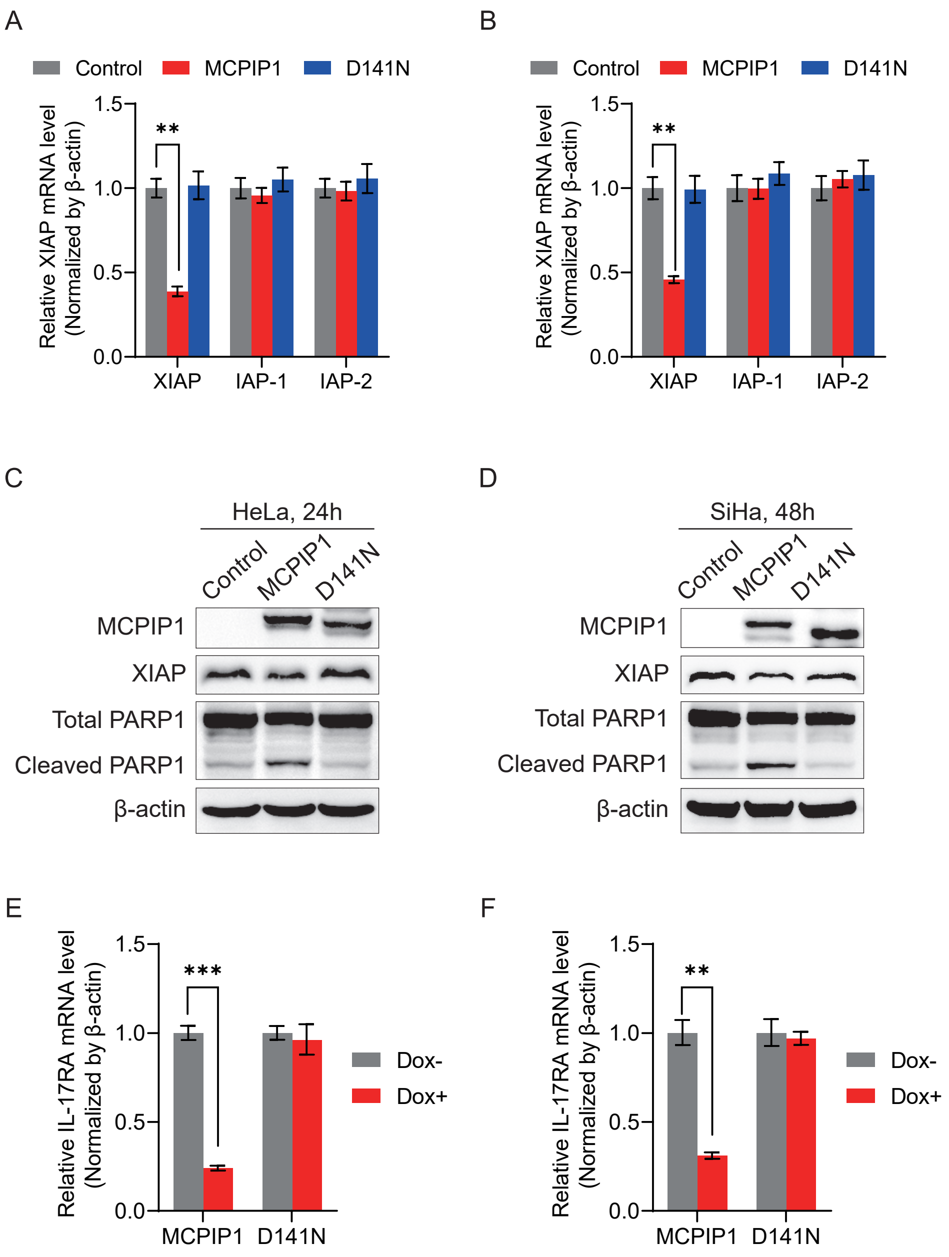

Supplementary Figure 2. MCPIP1, but not D141N, decreased XIAP mRNA and protein levels. HeLa and SiHa cells were infected with lentivirus of Control, MCPIP1, or D141N and treated with 1μg/ml doxycycline for 24 and 48 hrs, respectively. (**A, B**) The XIAP, IAP-1, and IAP-2 mRNA expression levels in HeLa (A) and SiHa (B) cells were determined by RT-qPCR analysis. (**C, D**) The protein expression levels of MCPIP1, XIAP, total PARP1, and cleaved PARP1 in HeLa (C) and SiHa (D) cells were detected by western blotting. β-actin was used as a loading control. (**E, F**) HeLa (E) and SiHa (F) cells were infected with lentivirus of MCPIP1 or D141N and treated with or without 1 μg/ml doxycycline for 24 and 48 hrs, respectively. Then, the IL-17RA mRNA levels were determined by RT-qPCR analysis.

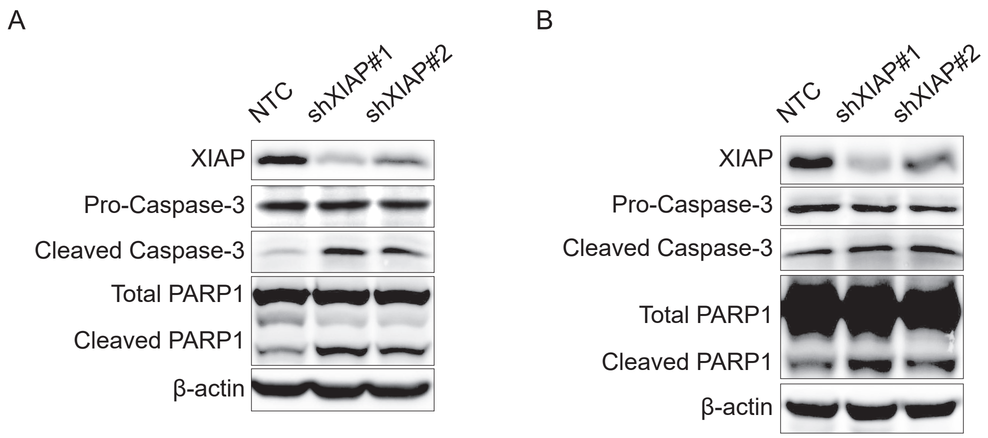

Supplementary Figure 3. Knockdown of XIAP promotes cervical cancer cell apoptosis. HeLa (**A**) and SiHa (**B**) cells were infected with the lentivirus of pLKO.1 (NTC) or shRNAs to XIAP (shXIAP#1 and shXIAP#2) for 48 hrs. Then, protein expression levels of XIAP, pro-caspase-3, cleaved caspase-3, total PARP1, and cleaved PARP1 were detected by western blotting. β-actin was used as a loading control.

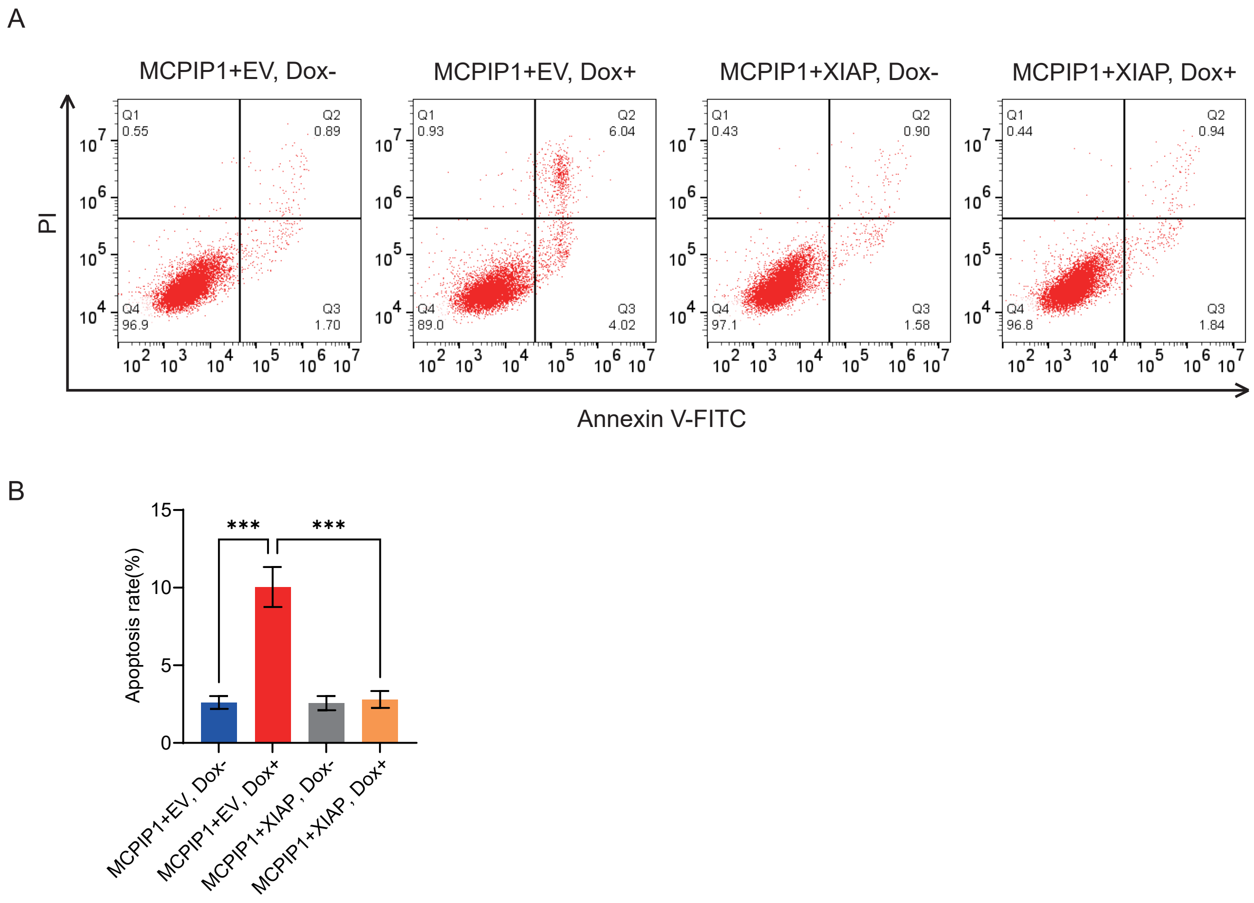

Supplementary Figure 4. XIAP impedes apoptosis-promoting function of MCPIP1 in SiHa cells. Flow cytometry assay was performed for apoptosis detection of SiHa cells co-infected with lentivirus of MCPIP1 and XIAP and treated with or without 1 μg/ml doxycycline for 48 hrs. Representative images (**A**) and statistical analysis based on three independent experiments (**B**) were shown.

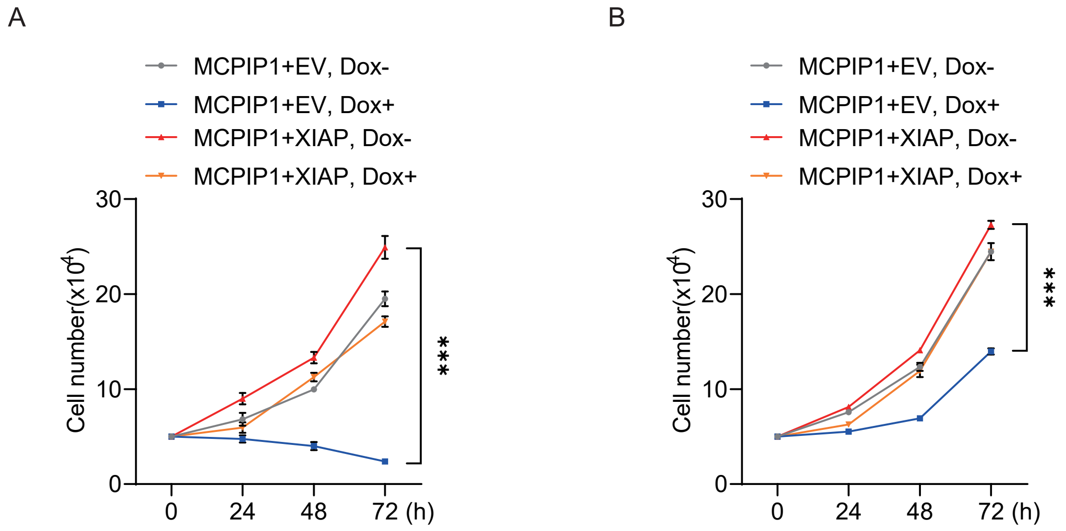

Supplementary Figure 5. XIAP overexpression rescued the proliferation of HeLa and SiHa cells impeded by MCPIP1. (**A**) HeLa cells were co-infected with lentivirus of MCPIP1 and EV or XIAP and treated with or without 1 μg/ml doxycycline for 24 hrs. Then, the number of living cells was counted at 0, 24, 48, and 72 hrs. (**B**) SiHa cells were coinfected with lentivirus of MCPIP1 and EV or XIAP and treated with or without 1μg/ml doxycycline for 48 hrs. Then, the number of living cells was counted at 0, 24, 48, and 72 hrs.

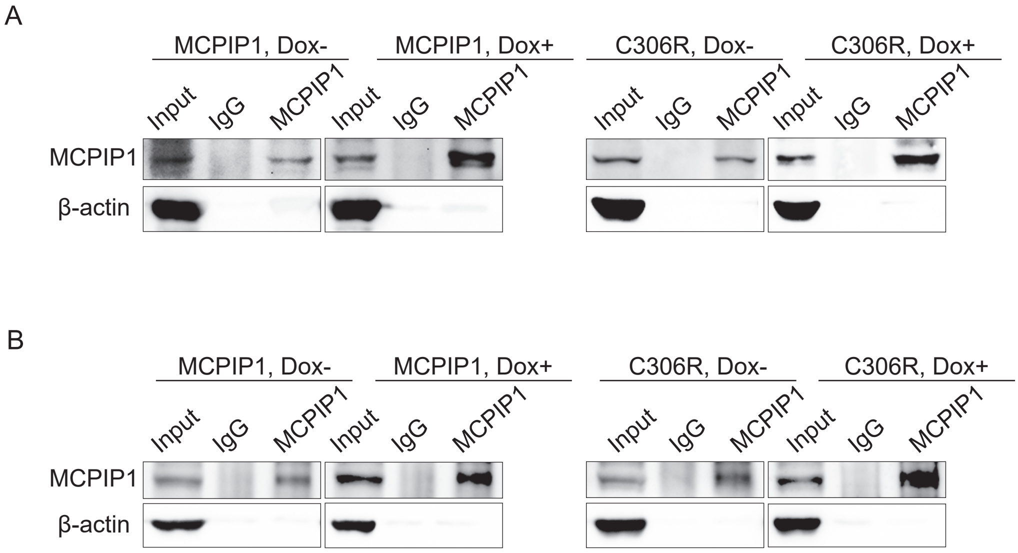

Supplementary Figure 6. The efficiency of MCPIP1 antibody for the enrichment of exogenous overexpressing MCPIP1 and C306R protein in RIP assay. Western blotting after RIP assay showed that MCPIP1 and C306R protein in HeLa (**A**) or SiHa (**B**) cell lysates were efficiently pulled down with the antibody to MCPIP1. β-actin was used as a negative control.

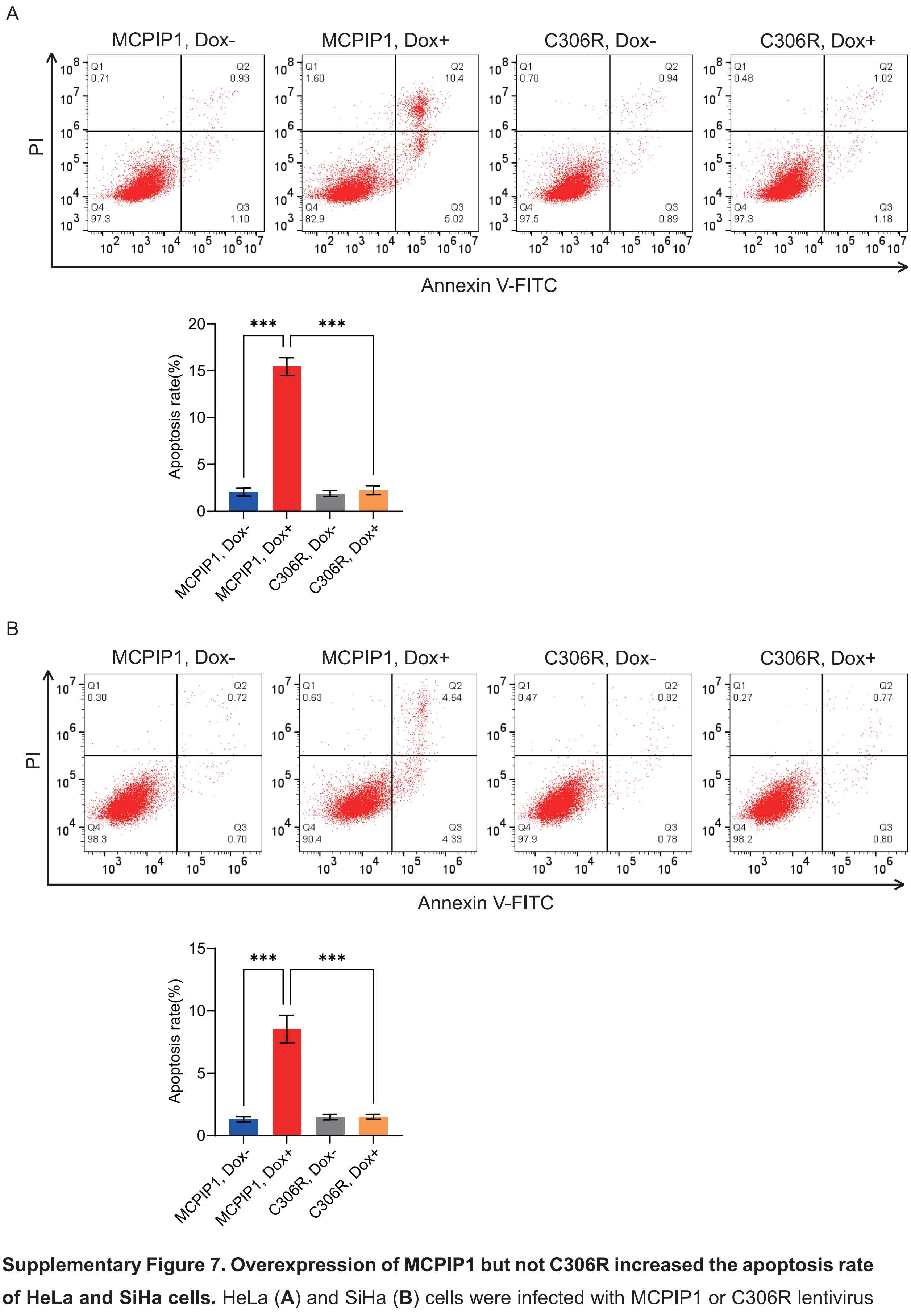

Supplementary Figure 7. Overexpression of MCPIP1 but not C306R increased the apoptosis rate of HeLa and SiHa cells. HeLa (**A**) and SiHa (**B**) cells were infected with MCPIP1 or C306R lentivirus and treated with or without 1 μg/ml doxycycline for 48 hrs. The apoptosis rate of cells was examined by flow cytometry assay. Representative images and statistical analysis based on three independent experiments were shown.

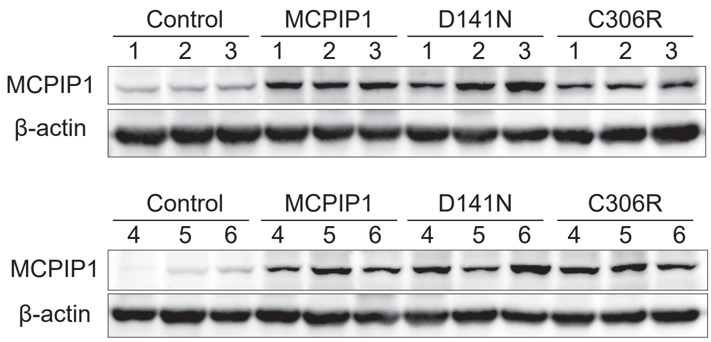

Supplementary Figure 8. MCPIP1 expression in tumor tissue of xenograft mouse model. Expression of MCPIP1 protein in the HeLa tumor after Doxycycline treatment was determined using Western blotting (n = 6). β-actin was used as an internal control.

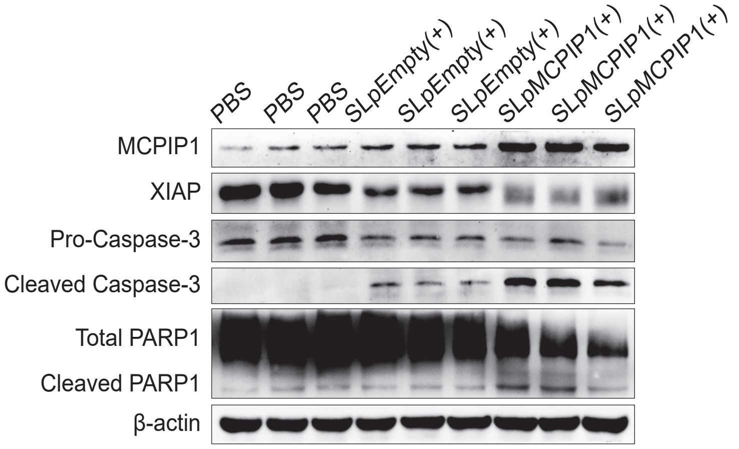

Supplementary Figure 9. MCPIP1-overexpressing bacteria enhances tumor cell death in xenograft mouse model. Immunoblots showing protein levels of MCPIP1, XIAP, pro-caspase-3, cleaved caspase-3, total PARP1, and cleaved PARP1 in Hela tumors at 3 days after L-arabinose induction (daily, starting at 3 dpi) (n = 3). β-actin was used as an internal control.

Supplementary Table 1. Primers for construction of MCPIP1 or XIAP overexpression and knockdown plasmids.

Oligo name	Sequence (5'-3')	Purpose
P_MCPIP1_For	GGAATTCATGAGTGGCCCCTGTGGAGA	Construction of pLVX-TetOne-MCPIP1 plasmid
P_MCPIP1_Rev	CGGGATCCTTACTCACTGGGGTGCTGGGACT	
P_XIAP_For	CCCTCGAGATGACTTTTAACAGTTTTGAAGG	Construction of pLVML-3xHA-XIAP plasmid
P_XIAP_Rev	GACTAGTTTAAGACATAAAAATTTTTTGC	
shMCPIP1#1_For	CCGGGCAGATGAAGGTGGACTTCTTCTCGAGAAG AAGTCCACCTTCATCTGCTTTTTG	Construction of pLKO.1-puro-shMCPIP1#1 plasmid
shMCPIP1#1_Rev	AATTCAAAAAAGCAGATGAAGGTGGACTTCTTCT CGAGAAGAAGTCCACCTTCATCTGC	
shMCPIP1#2_For	CCGGGAAATGCACCTATGGGATCAACTCGAGTTG ATCCCATAGGTGCATTTCTTTTTTG	Construction of pLKO.1-puro-shMCPIP1#2 plasmid
shMCPIP1#2_Rev	AATTCAAAAAAGCAGATGAAGGTGGACTTCTTCT CGAGAAGAAGTCCACCTTCATCTGC	
shXIAP#1_For	CCGGACACGTACTTGTGCGAATTATCTCGAGATAA TTCGCACAAGTACGTGTTTTTTG	Construction of pLKO.1-puro-shXIAP#1 plasmid
shXIAP#1_Rev	AATTCAAAAAACACGTACTTGTGCGAATTATCTCG AGATAATTCGCACAAGTACGTGT	
shXIAP#2_For	CCGGGCACTCCAACTTCTAATCAAACTCGAGTTTG ATTAGAAGTTGGAGTGCTTTTTG	Construction of pLKO.1-puro-shXIAP#2 plasmid
shXIAP#2_Rev	AATTCAAAAAGCACTCCAACTTCTAATCAAACTC GAGTTTGATTAGAAGTTGGAGTGC	
MCPIP1-D141N_For	GGTCATCAATGGGAGCAACGTGGCCATGAGCC	Construction of pLVX-TetOne-MCPIP1 (D141N) plasmid
MCPIP1-D141N_Rev	TGCTCCCATTGATGACCACTGGTCTCAGGTCG	
MCPIP1-C306R_For	AAGCAGCcgcGTCCCTATGGAAGGAAATGCAC	Construction of pLVX-TetOne-MCPIP1 (C306R) plasmid
MCPIP1-C306R_Rev	TAGGGACgcgGCTGCTTCCTGTGCTCCAAAGT	

Supplementary Table 2. Oligos for Real-time PCR.

Oligo name	Sequence (5'-3')	Purpose
XIAP_For	TGGGGTTCAGTTTCAAGGAC	Real-time PCR for detection of XIAP mRNA
XIAP_Rev	CGCCTTAGCTGCTCTTCAGT	
IAP-1_For	GAGGAGAAGGAAAAACAAGCTG	Real-time PCR for detection of IAP1 mRNA
IAP-1_Rev	TCCAGGATAGGAAGCACACA	
IAP-2_For	GGGAAGAGGAGAGAGAAAGAGC	Real-time PCR for detection of IAP2 mRNA
IAP-2_Rev	TCCAGGATTGGAATTACACAAG	
IL-17RA-For	CTGCGACTCCTGGACCAC	Real-time PCR for detection of IL-17RA mRNA
IL-17RA-Rev	TCAGGTTTCGAGGGTGAATC	
β-actin_For	AGAAAATCTGGCACCACACC	Real-time PCR for detection of β-actin mRNA
β-actin_Rev	GGGGTGTTGAAGGTCTCAAA	
